# Understanding and addressing contraceptive stockouts to increase family planning access and uptake in Senegal

**DOI:** 10.1186/s12913-017-2316-y

**Published:** 2017-05-26

**Authors:** Leah Hasselback, Modibo Dicko, Claire Viadro, Soussaba Ndour, Oumy Ndao, Jennifer Wesson

**Affiliations:** 1 Formerly IntraHealth International, Sacré Cœur Cité Keur Gorgui, derrière siège, SONATEL Lot R73, Dakar, Senegal; 2 IntraHealth International, Sacré Cœur Cité Keur Gorgui, derrière siège, SONATEL Lot R73, Dakar, Senegal; 3 IntraHealth International, 6340 Quadrangle Drive, Suite 200, Chapel Hill, NC 27517 USA

**Keywords:** Supply chain management, Stockouts, Contraceptives, Professionalization

## Abstract

**Background:**

Senegal’s government has pledged to reduce contraceptive stockouts, which have been frequent in public sector health facilities. An innovative distribution system called the Informed Push Model (IPM) addresses supply chain obstacles through direct regional-to-facility delivery of contraceptives and use of private sector logistics operators. Following promising pilot results, Senegal’s Ministry of Health and Social Action committed to a three-year (2013–2016) expansion of IPM to all public health facilities nationwide.

**Methods:**

From August 2014–July 2016, IPM’s six logisticians made 29,319 visits to restock public sector health facilities. During these regular facility visits, the logisticians conducted a physical inventory to flag contraceptive stockouts (no usable stock of any single method available) and asked facility staff to identify the primary reason for documented stockouts. Our descriptive study examines stockout trends over the course of IPM scale-up. We also describe trends in contraceptive consumption over the three-year period using facility-level data collected by the logisticians.

**Results:**

Contraceptive consumption rose by 91% over 35 months in the first three IPM regions, and by 118% in the next five regions (over 26 months). After scale-up to 1,394 health facilities, nationwide consumption rose by 48% over one year. On average, logisticians documented stockouts at fewer than 2% of facility visits. In comparison, two pre-IPM studies in 2011 identified stockouts of selected modern contraceptives at 60–70% of facilities visited, with 84% of clients reporting stockouts in the past year. Six factors (including consumption spikes, IPM-preventable causes, and community outreach) explained most remaining stockouts.

**Conclusions:**

IPM has been highly successful in ensuring full availability of contraceptives across regions and health facilities. The model also has facilitated the flow of essential data on consumption and stockouts from facilities up to district, regional, and central-level managers. These achievements highlight the relevance of professionalizing supply chain management while continuing to mitigate stockouts through enhanced stakeholder communication and improved training, coaching, and supervision of third-party logistics operators. Supply reliability is critical in shaping demand for and regular use of contraception. The government is transitioning the IPM to full management by the National Supply Pharmacy.

## Background

Senegal has high unmet need for family planning [1, 2]. According to the 2015 Demographic and Health Survey (DHS), one-fourth (25%) of reproductive-age women in union who wanted to delay their next birth or stop childbearing reported not using contraception, and the modern contraceptive prevalence rate (mCPR) for women in union was just 21% [2]. The lack of consistent access to family planning—which can reduce unintended pregnancies and allow women to space desired pregnancies [3]—has contributed to Senegal’s high maternal mortality ratio. In 2013, there were 320 maternal deaths per 100,000 live births, which was more than twice the country’s unmet Millennium Development Goal target of 130 per 100,000 [4, 5]. Recognizing the critical impact of family planning on maternal mortality, the government of Senegal committed in 2012 to increase the country’s mCPR to 45% by 2020.

As part of its ambitious family planning goals, the government of Senegal also pledged to strengthen the family planning supply chain. Strong health logistics, including supply chain management, are essential for strengthening health systems [6]. When contraceptive stockouts occur—defined as the unavailability of “one or more contraceptive options that, routinely or based on policy, should be available at a health facility” [7]—they represent a family planning access barrier [8] and signal that the supply chain is not functioning effectively [9]. Contraceptive stockouts have been frequent in Senegal’s public sector health facilities, where 84% of Senegalese women go when seeking modern methods of contraception. A baseline survey conducted by Senegal’s Urban Reproductive Health Initiative (URHI) in 2011 found that large proportions of public sector facilities (*n =* 153) in six urban areas had often experienced stockouts of key contraceptive methods over the past 12 months, including combined pills (over 70% of facilities), injectables (69%), emergency contraception (63%), and progestin-only pills (57%) [10]. The survey flagged facility-level contraceptive stockouts when a physical inventory found no usable stock of any single contraceptive method available at the facility, not including IUD and implant insertion kits. A separate URHI study of contraceptive stockouts in two districts of the Dakar region, which included interviews with women currently using contraceptives, found that 84% of clients reported a stockout of their preferred method in the past year [11].

The Dakar study estimated that at least 60% of the identified stockouts occurred despite stock availability at the national level, pointing to problems with in-country distribution [11]. In fact, as supply chain experts in Senegal and elsewhere have observed, the efficient and reliable distribution of commodities from central warehouses to lower-level health facilities is a frequently challenging and often neglected aspect of effective supply chain management [11–13]. In the past, Senegal used a highly complex order-based “pull” system. “Pull” supply chain strategies are driven by customer demand but tend to minimize stock on hand [14, 15]. Senegal’s system relied on health workers—who typically lack logistics management training—to accurately forecast, track, and order contraceptives and travel to regional or district warehouses to pick up supplies. However, these logistics duties impeded health workers’ ability to focus on their primary role of providing high-quality health and family planning services to clients. A study in Tanzania highlighted similar challenges when facilities shifted supply chain management duties to “non-pharmaceutical staff already overburdened with clinical care and administrative activities” [16].

Ensuring that health commodities reach the “last mile” is critical, but last-mile success hinges on the availability of timely and appropriate logistics and consumption data [13]. Senegal’s former pull system exacerbated supply chain management problems due to the lack of reliable and timely health facility data on contraceptive method preferences and consumption and the need for data to be funneled up from facilities to district and central-level managers. In addition, the pull system required that health facilities pay for supplies up front. When facilities use working capital to pay for commodities but are unable to replenish those funds until after clients purchase contraceptives, the result is either cash flow problems or situations where facilities channel limited funds toward non-contraceptive commodities that generate higher profit margins.

To address these problems, the government of Senegal expanded an innovative distribution system called the Informed Push Model (IPM). “Push” models require accurate forecasting but can encourage greater supply chain predictability by focusing on longer-term projections of customer demand [14, 15]. Senegal’s public-sector IPM adapts principles used in the commercial sector (such as vendor-managed inventory) to address common supply chain obstacles of transportation, quantification, availability of data, and financial flows. A number of other countries have implemented IPM variations, including the Delivery Team Topping Up and Informed Push systems in Zimbabwe [17, 18]; the Dedicated Logistics Systems in Mozambique [19]; the Direct Delivery and Information Capture model in Nigeria [20]; and the Informed Push Model in Togo.

In Senegal, the IPM streamlines the supply chain through direct regional-to-health-facility delivery of contraceptives, effectively bypassing the district level. To this end, third-party private sector logistics operators engaged through performance-based contracts collect facility-level consumption data and distribute contraceptives to facilities in a timely, consistent manner. During their delivery visits, the logistics operators carry out four sets of activities: (1) deliver contraceptives to each facility on a monthly basis; (2) count stock levels and top up commodities to a level that equates to approximately a three-month supply (based on consumption during the prior three months); (3) collect data to forecast future delivery quantities; and (4) work with facility staff to identify changing needs or upcoming activities that might lead to short- and long-term changes in the quantity of stock required (e.g., demand generation campaigns by community-based organizations) [14].

Whereas the pull system required up-front payment, the IPM charges facilities post-consumption. Both the government and the IPM have cost-recovery policies that require clients to pay a fee for all contraceptives other than male and female condoms. Health facilities keep some of these revenues and pay out portions of the balance to districts, the National Supply Pharmacy, and the Ministry of Health and Social Action, respectively. Facilities use their retained cost-recovery funds to pay for the contraceptives consumed, eliminating cash flow difficulties. An electronic logistics management information system allows for mobile data collection and real-time data on deliveries, consumption, and cost-recovery.

As reported elsewhere, pilot implementation of the IPM in 2011–2012 dramatically reduced contraceptive stockouts at 140 health facilities (health centers and health posts) across the Dakar region to less than 2% in just 12 months [11]. By way of comparison with other countries in sub-Saharan Africa, in 11 of 12 countries, the percentage of health facilities reporting some type of stockout in the past six months as of 2013 ranged from one-fifth (20%) to nearly all (97%) [9]. Given the IPM’s extremely promising pilot results, Senegal’s Ministry of Health and Social Action—together with IntraHealth International (an international nongovernmental organization), the Bill & Melinda Gates Foundation, and *Merck for Mothers*—committed to a three-year, phased national IPM expansion. The IPM initially was scaled up to 559 health facilities in three regions (2012–2013) and later expanded to 1,394 public facilities in all 14 regions of Senegal over a three-year period (2013–2016).

This paper has two aims. First, we describe trends in contraceptive consumption and observed reductions in stockouts following scale-up of the IPM. Using the IPM’s robust dataset—which includes information about facility-level causes of stockouts—we also examine stockouts that persist even after most major supply chain obstacles have been addressed. This information can help identify strategies to further anticipate, reduce, and prevent stockouts.

## Methods

During monthly delivery and inventory visits to health facilities, the IPM’s trained logistics professionals collected two broad categories of stockout data. First, the logisticians conducted a physical inventory of contraceptive methods to identify stockouts. Currently, facility inventory should include ten methods: combined and progestin-only pills, standard injectables, the Sayana® Press subcutaneous injectable (in scale-up), implants, intrauterine devices (IUDs), male and female condoms, CycleBeads®, and emergency contraception [14]. Logisticians documented a facility-level stockout if the physical inventory found no usable stock of any single contraceptive method available. When stockouts were identified, the logisticians asked facility staff to identify the primary reason for the stockout. Only one reason could be given per stockout occurrence.

The logistics operators entered all data into tablets and also recorded information on paper forms, with one copy left at the facility and one copy sent to the IntraHealth office in Dakar. Subsequently, the logistics operators compiled the stockout information in monthly reports. IntraHealth staff reviewed the data for errors and outliers and carried out quarterly data audits. When the logisticians’ monthly reports failed to provide information about specific stockouts, IntraHealth staff followed up with the logisticians and/or facility staff to ascertain the primary reason for the stockout.

## Results

We present three sets of results pertaining to the national scale-up of the IPM in Senegal. First, to set the stage for understanding the implications of eliminating stockouts, we describe the trend toward increased contraceptive consumption that followed IPM expansion. Second, we review stockout data and achievements, and third, we consider available information about the causes of lingering stockouts.

### Contraceptive consumption

**Fig. 1:**
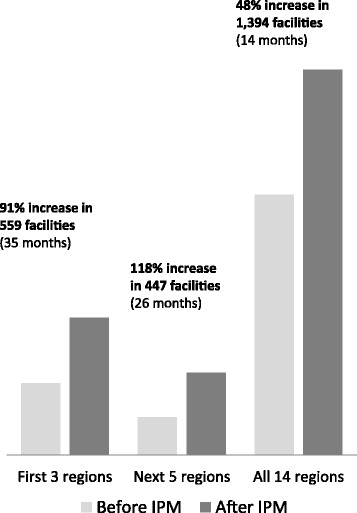
Number of contraceptives consumed following IPM implementation in 14 regions of Senegal, all methods combined (2013-2016)

Consumption of contraceptive products increased considerably following IPM implementation and scale-up (Fig. 1). In the first three regions in which the IPM was implemented (559 health facilities), total consumption increased by 91% over 35 months (from 93,421 contraceptives in July 2013 to 178,502 in May 2016). In the next five regions (447 facilities), contraceptive consumption increased by 118% over the 26-month period from April 2014 to May 2016 (from 49,133 to 107,109 contraceptives). Finally, nationwide consumption increased by 48% (from 337,698 to 499,824 contraceptives) over the 14-month period from April 2015 to May 2016 after full IPM scale-up to 1,394 facilities in all 14 regions.

### Stockout patterns

Six third-party logistics operators made a total of 29,319 monthly deliveries to health facilities over a 24-month period ending in July 2016. Prior to full scale-up (August 2014), the logisticians made deliveries to 840 facilities; by March 2015, when IPM scale-up in the 14 regions was fully achieved, the logistics operators were reaching 1,394 facilities. On average, the logisticians made one delivery per month and per facility, with the exception of facilities that become inaccessible during the rainy season; in those instances, the logistics operators supplied the latter with extra “winter”/rainy season stocks.

**Fig. 2:**
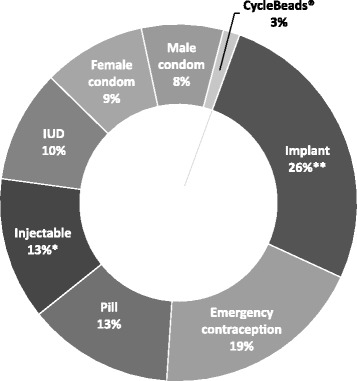
Contraceptive stockouts (%), by method (August 2014-July 2016). *Sayana® Press (10.7%) and Depo-Provera (2.2%). **Implanon implant (13.4%) and Jadelle implant (12.9%)

The two URHI studies that examined stockouts prior to implementation of the IPM in 2011 documented stockouts of modern contraceptives such as injectables and pills at 60 to 70% of facilities visited [10] and found that over 80% of clients had been unable to obtain their preferred method in the past year [11]. After IPM scale-up, the logistics operators reported stockouts (of any contraceptive method) during only 551 (1.9%) of the 29,319 facility visits. Over one-third of the stockouts (36%) were of long-acting reversible contraceptives (LARCs), including the Jadelle and Implanon implants and the IUD (Fig. 2). Emergency contraception represented another 19% of stockouts. The quarterly stockout rate varied from a low of under 1% to a high of over 4% (Table 1). The noticeable increase in stockouts in August to October 2015 coincided with the testing in five regions of three different scenarios for integrating new products into the IPM.

**Table 1:** Contraceptive stockouts in health facilities, by quarter (August 2014–July 2016)

Quarter	Number of facilities visited per quarter	Number of facilities with stockouts	Average quarterly stockout rate
August-October 2014	2,653	35	1.3%
November 2014-January 2015	2,963	27	0.9%
February-April 2015	3,551	63	1.8%
May-July 2015	3,978	66	1.7%
August-October 2015	3,756	154	4.1%
November 2015-January 2016	4,099	85	2.1%
February-April 2016	4,134	38	0.9%
May-July 2016	4,185	83	2.0%
Total	29,319	551	1.9%

### Stockout causes

Despite the dramatic decline in contraceptive stockouts after IPM implementation, 551 stockouts occurred over the 24-month period under study. Our review of each individual stockout occurrence identified six broad categories of stockout triggers accounting for two-thirds of stockouts (Table 2). The remaining one-third of stockouts occurred for a variety of other or unknown reasons.

**Table 2:** Reasons for contraceptive stockouts in 14 regions of Senegal, August 2014 to July 2016 (*N =* 551 stockouts)

Stockout causes	Number of instances of stockouts	Percentage
Spikes in consumption	149	27.0%
IPM-preventable	118	21.4%
Outreach	76	13.8%
Nonpayment	13	2.4%
New health facility	11	2.0%
Other	184	33.4%
Total	551	100%

#### Spikes in consumption

About one-fourth (27%) of the facility visits at which stockouts were noted arose due to atypical or unanticipated increases in the quantity of contraceptives consumed following a previously stable period of consistent consumption. Nearly all method types experienced some unexpected spikes in consumption.

#### IPM-preventable

One in five (21%) of the stockouts were due to avoidable errors by the logistics operators and/or IPM staff. Ordinarily, with adequate training, coaching, and supervision, the private third-party logistics operators should be able to prevent mistakes such as failing to substitute current products for those about to expire, failing to deliver or miscalculating delivery quantities, or distributing the entire stock of a product to one facility.

#### Outreach

Almost 14% of stockouts were due to community outreach programs. This type of stockout occurred when the outreach activities were not planned in advance or were not communicated to the logisticians, resulting in delivery of insufficient product to cover both the outreach activities and routine facility-based service needs.

#### Nonpayment

Clients pay a fee for all contraceptives except male and female condoms as part of the government’s cost-recovery system. If a facility has not paid its bills, district managers can request that the facility not be resupplied. However, this type of stockout was observed only a handful of times.

#### New facilities

IPM estimates delivery quantities based on a facility’s past consumption patterns. When a new facility opens, stockouts may occur during the early months until consumption stabilizes and can be predicted more accurately.

#### Other/unknown reasons

Contraceptive resupply takes place in a dynamic and exception-filled environment. Miscellaneous reasons accounting for some stockouts included staff being absent or otherwise unable to receive deliveries; substitution of one type of implant for another; absence of timely consumption data for correct forecasting; and health facility repair or renovations. Although each individual cause occurred only a limited number of times, altogether they represented the biggest cause of lingering stockouts (33%).

Table 2 omits “insufficient upstream stock” among the causes of stockouts. We excluded upstream shortages from our analysis because inadequate upstream supplies at either the National Supply Pharmacy or the regional supply depots are entirely independent of the IPM sphere of operations. IPM has no means to act on such shortages other than to draw the attention of National Supply Pharmacy management to the problem. Moreover, given the stringent definition of a stockout (no usable stock of any single contraceptive method available), a stockout of a single contraceptive commodity at the level of the National Supply Pharmacy will trickle down to create stockouts in all of the country’s health facilities, resulting in a temporary stockout rate of 100% even when all other products are available.

## Discussion

Prior to the IPM, Senegal’s public sector health facilities did not compile facility-level data on contraceptive stockouts. The only previously available stockout data, gathered by the two regional URHI studies carried out in 2011 prior to IPM implementation, documented widespread stockouts of several modern methods (60–70%) [10], a finding corroborated through interviews with current contraceptive users, over four-fifths (84%) of whom had experienced a recent stockout [11]. Similarly, in other sub-Saharan African countries, the percentage of health facilities reporting stockouts in the past six months is often 50% or greater [9]. IPM expansion to all 14 regions of Senegal changed this picture dramatically, with contraceptive stockouts documented at only 1.9% of facility visits over the two-year period from 2014 to 2016. Alongside the steep decline in stockouts, contraceptive consumption (all methods combined) rose by 91% over a three-year period in the first three regions enrolled in IPM. After national IPM scale-up was achieved in March 2015, national consumption rose by 48% over the one-year period from April 2015 to May 2016. These favorable trends confirm that supply reliability plays a critical role in shaping demand for and regular use of contraceptive methods [21].

IPM also achieved significant gains in routine availability of the contraceptive data that allow for accurate forecasting. Under IPM, delivery teams capture real-time consumption data each month in all health facilities across the country; these data are then made available to managers at all levels of the health system on a monthly basis through an Internet platform. This process results in more accurate forecasting of needs compared to prior forecasting, which was derived solely from the quantities delivered from regional pharmacy depots.

Facility managers, family planning service providers, and pharmacy and storekeeping staff all have important roles to play in eliminating contraceptive stockouts. At the same time, professionalizing supply chain management is likely a necessary step to strengthen the health supply chain and, ultimately, health system performance. A study conducted in Zambia showed that professionalization of district logistics management almost doubled malaria drug availability and could cut down child mortality due to malaria by 37% [22]. Through its reliance on private third-party logistics providers, Senegal’s IPM has decreased the technical logistics burden shouldered by the health workers who provide family planning services [23, 24]. Professionalized logistics managers have the advantage of being able to “identify and implement the best solutions in given contexts, to innovate and to adapt through time and as circumstances change” [6]. In short, Senegal’s logisticians serve as the “missing link” that bridges higher-level management functions and frontline service delivery [6].

The introduction and scale-up of IPM successfully addressed most major supply chain obstacles, but some stockouts persisted. Our analysis revealed a wide variety of underlying reasons for lingering stockouts, including unanticipated spikes in consumption, logistician error, and unplanned outreach activities, among others. Most of these stockouts could probably have been prevented through better coordination between logisticians, facility and district managers, and others. Ongoing communication and coordination is essential to ensure that health facilities actively anticipate potential stockouts rather than responding to stockouts well after the fact or ignoring stockouts and simply awaiting the next delivery. For example, although outreach activities usually are planned in advance, if outreach organizers do not let health facilities know about the planned activities, the facilities and logisticians cannot anticipate the subsequent effects on demand for methods. Improved training and coaching and rigorous supervision of private third-party operators can also bring about a substantial reduction in stockouts. On the other hand, our analysis points to the ad hoc and unpredictable nature of a small proportion of stockouts, some of which may be impossible to completely prevent.

Senegal’s high-level government commitment to family planning and IPM was instrumental in achieving the tremendous increase in contraceptive consumption and decreased stockouts documented following IPM scale-up [25]. Regular stakeholder meetings can solidify policy-makers’ commitment, while also ensuring that all stakeholders recognize their respective roles, have the technical skills required, and understand how their actions may influence stock availability at the facility level. To be most effective, such meetings should be led by the government agencies responsible for the supply chain and should include individuals with the authority to make decisions and implement proactive measures to reduce stockouts.

Due to the success of the IPM, the Ministry of Health and Social Action has directed that the National Supply Pharmacy take over full management of the system, including continuing to use private operators and incorporating other essential health commodities in addition to contraceptives [25]. A National Technical Committee established by the Minister of Health and Social Action is overseeing this transition in collaboration with the National Supply Pharmacy, and the transition is expected to be complete by early 2018. As of 2014, estimates placed the operating cost of the IPM at approximately $500,000, but additional analyses highlighted the potential for cost savings across Ministry of Health programs as the commodity distribution system becomes less fragmented [11]. Moreover, both the government and the IPM have cost-recovery policies whereby clients pay a fee for nearly all contraceptive products. These revenues form the basis for a self-financing mechanism that should allow the IPM to move toward sustainability. A recent review of the literature on sustainability of health interventions in sub-Saharan Africa noted that “working within existing resources” is a key facilitator of sustainability after withdrawal of external funding and assistance [26]. The practices and cost- recovery model developed under the IPM are likely to have considerable relevance for other countries in sub-Saharan Africa functioning with similar health system financing mechanisms.

### Limitations

It is clear that a variety of factors have converged to encourage the favorable trends in contraceptive consumption witnessed in Senegal over the past several years, including but certainly not limited to the successful rollout and scale-up of the IPM. Other important contributing factors include consistently strong government and Ministry of Health attention to and support for family planning and reproductive health [25, 27]; extensive commitments from bilateral donors such as the U.S. Agency for International Development [28]; successful initiatives to involve imams and other religious leaders in addressing religious barriers to family planning [29]; and demand creation activities [27].

To our knowledge, our detailed analysis of stockout causes is unique. Ironically, IPM’s dramatic success in reducing stockouts means that there were relatively few persistent stockouts to examine, limiting our ability to make inferences about stockout patterns over time. Because the logisticians derived the reasons for stockouts from health workers’ subjective perceptions of what happened, the IPM team conducted quarterly data audits to balance out the limitations of these subjective impressions.

Stockouts can be studied in different ways. Given the availability of logistics data, the IPM employed a rigorous definition of stockouts (i.e., no usable stock of any single method available) that addressed product outages primarily as an inventory issue. Qualitative studies in other settings have gone beyond supply chain issues to examine the “human face” of contraceptive stockouts, analyzing clients’ and providers’ perceptions of stockouts, client coping strategies, and adverse consequences of stockouts [30, 31]. These types of questions were beyond the scope of the IPM’s supply chain management focus and data.

## Conclusions

Senegal’s Informed Push Model has provided a technical supply chain solution that successfully addresses problems with transportation, quantification, and financial flows, resulting in dramatically increased consumption and reduced stockouts. However, because health facilities are dynamic environments, sometimes they may require more than a purely technical fix. Our detailed analysis of stockout causes provides information on how to further reduce stockouts once obvious system failures have been corrected. Addressing other factors contributing to stockouts requires improved coordination, communication, and health systems management. Good-quality commodities can only be available at the right time and in the right quantity when service delivery and supply chain systems are integrated, and all actors perform their designated roles.

## Abbreviations

DHS: Demographic and Health Survey
IPM: Informed Push Model
IUD: Intrauterine device
LARCs: Long-acting reversible contraceptives
mCPR: Modern contraceptive prevalence rate
URHI: Urban Reproductive Health Initiative
